# Attitudes toward depression among Japanese non-psychiatric medical doctors: a cross-sectional study

**DOI:** 10.1186/1756-0500-5-441

**Published:** 2012-08-16

**Authors:** Tsuyuka Ohtsuki, Manami Kodaka, Rumi Sakai, Fuminobu Ishikura, Yoichiro Watanabe, Anthony Mann, Mark Haddad, Mitsuhiko Yamada, Masatoshi Inagaki

**Affiliations:** 1 Center for Suicide Prevention, National Institute of Mental Health, National Center of Neurology and Psychiatry, Kodaira, Tokyo, Japan; 2 Hyogo Mental Health Center, Kobe, Hyogo, Japan; 3 Department of Functional Diagnostic Science, Division of Health Science, Osaka University Graduate School of Medicine, Suita, Osaka, Japan; 4 Osaka Association of Psychiatric Clinics, Osaka, Japan; 5 Department of Health Services and Population Research, Institute of Psychiatry at King's College London, London, UK; 6 School of Health Sciences, City University London, London, UK; 7 Department of Neuropsychopharmacology, National Institute of Mental Health, National Center of Neurology and Psychiatry, Kodaira, Tokyo, Japan; 8 Section of Medical Research for Suicide, Center for Suicide Prevention, National Institute of Mental Health, National Center of Neurology and Psychiatry, 4-1-1 Ogawahigashimachi, Kodaira, Tokyo, 187-8553, Japan

## Abstract

**Background:**

Under-recognition of depression is common in many countries. Education of medical staff, focusing on their attitudes towards depression, may be necessary to change their behavior and enhance recognition of depression. Several studies have previously reported on attitudes toward depression among general physicians. However, little is known about attitudes of non-psychiatric doctors in Japan. In the present study, we surveyed non-psychiatric doctors’ attitude toward depression.

**Methods:**

The inclusion criteria of participants in the present study were as follows: 1) Japanese non-psychiatric doctors and 2) attendees in educational opportunities regarding depression care. We conveniently approached two populations: 1) a workshop to depression care for non-psychiatric doctors and 2) a general physician-psychiatrist (G-P) network group. We contacted 367 subjects. Attitudes toward depression were measured using the Depression Attitude Questionnaire (DAQ), a 20-item self-report questionnaire developed for general physicians. We report scores of each DAQ item and factors derived from exploratory factor analysis.

**Results:**

We received responses from 230 subjects, and we used DAQ data from 187 non-psychiatric doctors who met the inclusion criteria. All non-psychiatric doctors (n = 187) disagreed with "*I feel comfortable in dealing with depressed patients' needs,*" while 60 % (n = 112) agreed with "*Working with depressed patients is heavy going.*" Factor analysis indicated these items comprised a factor termed "*Depression should be treated by psychiatrists*" - to which 54 % of doctors (n = 101) agreed*.* Meanwhile, 67 % of doctors (n = 126) thought that nurses could be useful in depressed patient support. The three factors derived from the Japanese DAQ differed from models previously derived from British GP samples. The attitude of Japanese non-psychiatric doctors concerning whether depression should be treated by psychiatrists was markedly different to that of British GPs.

**Conclusions:**

Japanese non-psychiatric doctors believe that depression care is beyond the scope of their duties. It is suggested that educational programs or guidelines for depression care developed in other countries such as the UK are not directly adaptable for Japanese non-psychiatric doctors. Developing a focused educational program that motivates non-psychiatric doctors to play a role in depression care is necessary to enhance recognition and treatment of depression in Japan.

## Background

Depression is a common mental disorder associated with impaired quality of life. Depression was the leading cause of disability in middle- and high-income countries in 2004, and will become the leading cause of disability worldwide in 2030 [1]. However, it is reported that many depressed patients may not be given appropriate care in many countries, including Japan [2,3].

In the Japanese medical system, most doctors have specialties, such as internal medicine and surgery, and treat specific illnesses. Patients select and consult such specialists freely and directly based on their own judgment and preference. However, in the case of psychiatric disorders, such as depression, patients do not always consult psychiatrists directly. Reasons for this may include a lack of perception of their symptoms as those of depression or stigma associated with psychiatric disorders and psychiatric care [2]. Furthermore, symptoms such as insomnia and fatigue are frequent in depression, and may lead patients to consult non-psychiatric doctors with specialties in physical illness [4]. For these reasons, non-psychiatric doctors play an important role in identifying depressed patients and introducing them to appropriate treatment.

Under-recognition of depression is common in non-psychiatric settings, such as primary care, in many countries [5]. In our previous study, we found that physicians in a general internal medicine outpatient clinic in Japan recognized few depressed patients, even though the prevalence of depression was high [6]. One reason for the low recognition rate by non-psychiatric doctors may be their attitudes toward depression. Doctors’ attitudes toward depression in countries with a primary care system and general practitioners (GPs) have been surveyed and reported in the UK [7-9] and elsewhere [10-12]. However, there has been no such study in Japan. Information on the attitudes of non-psychiatric doctors toward depression in Japan is necessary to develop an educational intervention to facilitate the role of doctors in depression care and to build and optimize depression care settings in the Japanese medical system.

A previous study has shown that physicians who attended educational lecture were significantly more likely to change their clinical behavior if they indicated an intent to change prior to the lecture [13]. We hypothesized doctors who voluntarily accessed an educational opportunity would already have relatively high motivation to treat patients with depression. This population would be a primary target of future educational interventions.

Therefore, in this study, we surveyed the attitudes of non-psychiatric doctors who voluntarily accessed an educational opportunity in Japan. We also discuss their attitudes and compare the results with those from studies carried out previously in the UK.

## Methods

### Participants

The inclusion criteria of participants in the present study were as follows: 1) Japanese non-psychiatric doctors and 2) attendees in educational opportunities regarding depression care. We recruited the participants who satisfied both criteria. We conveniently approached two populations: 1) a workshop to depression care for non-psychiatric doctors and 2) a general physician-psychiatrist (G-P) network group.

We surveyed attitudes of 217 subjects who voluntarily attended a workshop regarding depression care. The workshop was sponsored jointly by the local government and a medical association in the Kansai area in January 2009. Our survey was performed before the lecture. In addition, we contacted 150 of the 210 members of a G-P network group in the Kansai area, who had agreed to participating in this survey. The G-P network is a voluntary group established for increasing general physicians’ understanding of mental health, enhancing collaborations between general physicians and psychiatrists, and facilitating patient referrals among doctors in the network. The members had been attending educational meetings regarding depression care two to three times a year. In June 2008, the survey was performed to the group members by mail with a stamped and self-addressed envelope for return. As a result, we contacted 367 subjects in total. For convenience, we refer to doctors with non-mental health specialties as “non-psychiatric doctors.”

This study was approved by the Ethics Committee of the National Center of Neurology and Psychiatry in Japan [xxxx-030 (19-7-JI1)]. The need for written informed consent was waived by the Ethics Committee. We informed the aims and methods of the present survey, including risks and benefits to the participants using written documents. Participants were asked to complete the questionnaire anonymously assuming that they agreed with the study aims and methods.

### Measures

#### Background characteristics

All participants were asked to indicate their age, sex, and specialty in the questionnaire.

#### Attitude toward depression

We measured attitudes toward depression using the Depression Attitude Questionnaire (DAQ) [7]. We decided to use the DAQ because we also aimed to compare the attitudes of Japanese non-psychiatric doctors to those previously reported in the UK. The DAQ was developed to measure GPs’ attitudes toward depression in the UK [7]. This self-report questionnaire consists of 20 items and was constructed based on three themes: the conceptual model of the responder, the respondents’ value judgments, and their practical response. Answers are marked on a 100-mm visual analogue scale (VAS) ranging from “strongly disagree” (0 mm) to “strongly agree” (100 mm). In addition to investigating the attitudes toward depression [7], the DAQ has also been used to assess the effectiveness of teaching GPs skills in brief cognitive behavior therapy [14], and to explore how family physicians perceive recognition, diagnosis, and depression management [11].

The DAQ was translated into Japanese by two psychiatrists, a psychiatric social worker, and an internist. As stated in the Background section, most doctors have specialties and treat specific illness in Japan. Therefore, we added a following explanation to the Japanese version of the DAQ: *In the following statements, whenever the term “general practice” is used, substitute it with your own specialized field of medicine when answering*.

We administered the preliminary Japanese version of the DAQ to 10 non-psychiatric doctors and 5 psychiatrists. According to their recommendations, we revised the terms “comfortable” in Item 9 and “working with” in Item 13.

The revised Japanese version was then back-translated into English by independent native English translators who were fluent in Japanese. Discrepancies emerging between this back-translated version and the original version were discussed with the developer of the original version, and the Japanese version was revised again. After this process, the Japanese DAQ was finalized (Additional file 1).

### Analysis

#### Summary of attitudes

As our primary outcomes, participants’ DAQ responses were classified into one of three categories based on previous studies [9,15]: “disagree” (0-33.3), “neutral” (33.4-66.6), and “agree” (66.7-100). In addition, we calculated the mean score of each item in accordance with previous studies, which enabled us to compare these with findings from other samples and settings [7,8].

We supplementarily performed an exploratory factor analysis of all 20 DAQ items. The factor solution was determined on the basis of an eigenvalue in excess of 1.0 and a scree plot, and a varimax rotation was applied to the extracted factors. We excluded items with a weak loading on any factor (< 0.35) and repeated analyses under the same conditions until all item loadings exceeded 0.35. Reliability coefficients were calculated using Cronbach’s alpha values for the factors derived from exploratory factor analysis. We excluded items with low corrected item-total correlation and repeated the analysis under the same conditions.

To show distributions of agreement in each factor, we calculated the mean score of items constructing each factor derived from the factor analysis in accordance with previous studies [8,15]. We defined this as the “factor score.” If item loadings had negative values, subtracted values from 100 were used in the calculation. Each factor score was divided into three levels of agreement, similar to the item score categories.

#### Comparison to the GP factor models in the UK

As subanalyses, we performed confirmatory factor analyses to assess the degree of fit to models previously reported. We calculated several fit indices, including chi-square, goodness of fit index (GFI), adjusted goodness of fit index (AGFI), comparative fit index (CFI), root mean square error of approximation (RMSEA), and the Akaike information criterion (AIC) by maximum likelihood estimation method for comparison with the four-factor model [7] and the three-factor model [9] derived from GP surveys.

#### Data analysis

We performed statistical analyses using SPSS version 17.0 and Amos version 17.0 (SPSS Japan Inc.). We did not correct for multiple comparisons because the primary outcome of this study was descriptive data regarding attitudes, and comparisons were secondary analyses to add explanatory information regarding the main result.

## Results

### Participant characteristics

We received questionnaires from 230 subjects (62.7 %) out of 367. Of these 230 respondents, we excluded six doctors whose specialties were unknown. Of the remaining 224 doctors, 21 psychiatrists were excluded. Sixteen non-psychiatric doctors had deficits in some items of the DAQ. As a result, we used DAQ data from 187 non-psychiatric doctors.

The median age of all non-psychiatric doctors was 53 (range 32-82) years and 78.1 % were male (Table 1). The majority of non-psychiatric doctors specialized in internal medicine (68.1 %).

**Table 1:** Participant characteristics (n = 187)


Participant group		
G-P network group members	57	30.5 %
Workshop attendees	130	69.5 %
Sex^a^		
Male	143	78.1 %
Female	40	21.9 %
Median age (range) (years) ^a^	53.0 (32-82)	
Specialty^a,b^		
Internal medicine	124	68.1 %
Surgery	26	14.3 %
Both internal medicine and surgery	6	3.3 %
Other	26	14.3 %

^a^ Several participants did not provide responses.

^b^ The internal medicine group included general internal medicine, cardiovascular internal medicine, gastroenterological medicine, respiratory medicine, and neurology. The surgery group included general surgery, orthopedic surgery, gastroenterological surgery, and neurosurgery. Other specialties included pediatrics, obstetrics and gynecology, radiology, anesthesiology, otorhinolaryngology, dermatology, urology, proctology, dialysis, industrial physicians, and administrative posts in local governments.

### Attitudes toward depression

Non-response rate of each item was listed in Table 2. Categorized results in non-psychiatric doctors and the mean scores for each item were listed in Table 3. Items with “agree” scores from more than 50 % of the non-psychiatric doctors were: “*During the last 5 years, I have seen an increase in the number of patients presenting with depressive symptoms*” (Item 1), “*The practice nurse could be a useful person to support depressed patients*” (Item 12), “*Working with depressed patients is heavy going*” (Item 13), “*If depressed patients need antidepressants, they are better off with a psychiatrist than with a general practitioner*” (Item 17), and “*Psychotherapy for depressed patients should be left to a specialist*” (Item 19).

**Table 2:** **Non**-**response rate of each item**

**Item No.**	**non-response n (%)**	**Item No.**	**non-response n (%)**
1	5 (2.5)	11	8 (3.9)
2	5 (2.5)	12	7 (3.4)
3	7 (3.4)	13	7 (3.4)
4	8 (3.9)	14	14 (6.9)
5	7 (3.4)	15	6 (3.0)
6	6 (3.0)	16	10 (4.9)
7	7 (3.4)	17	5 (2.5)
8	6 (3.0)	18	9 (4.4)
9	8 (3.9)	19	6 (3.0)
10	9 (4.4)	20	7 (3.4)

**Table 3:** Percentage of responses in each agreement category and mean score for individual items

	**Agreement (%)**			**Mean score (SD)**
	**Agree**	**Neutral**	**Disagree**	
**Factor I: Depression should be treated by psychiatrists**	54.0	43.3	2.7	68.1 (17.2)
13 Working with depressed patients is heavy going.	59.9	33.7	6.4	67.1 (20.8)
17 If depressed patients need antidepressants, they are better off with a psychiatrist than with a general practitioner.	57.2	34.2	8.6	68.1 (22.3)
19 Psychotherapy for depressed patients should be left to a specialist.	62.0	27.8	10.2	69.1 (24.1)
**Factor II: Pessimism regarding depression treatments**	2.1	54.5	43.3	35.5 (13.4)
1 During the last 5 years, I have seen an increase in the number of patients presenting with depressive symptoms.	56.7	39.6	3.7	68.9 (20.1)
14 There is little to be offered to those depressed patients who do not respond to what GPs do.	4.8	35.8	59.4	30.4 (20.1)
15 It is rewarding to spend time looking after depressed patients.	44.4	47.1	8.6	62.4 (19.3)
16 Psychotherapy tends to be unsuccessful with depressed patients.	11.2	55.6	33.2	42.8 (19.9)
**Factor III: Prejudice regarding depression etiology and pathology**	5.9	77.0	17.1	46.9 (14.6)
6 It is possible to distinguish two main groups of depression: one psychological in origin and the other caused by biochemical mechanisms.	29.4	57.2	13.4	55.7 (21.4)
7 Becoming depressed is a way that people with poor stamina deal with life difficulties.	12.3	34.2	53.5	35.1 (24.3)
8 Depressed patients are more likely to have experienced deprivation in early life than other people.	17.6	55.1	27.3	45.5 (20.8)
10 Depression reflects a characteristic response in patients which is not amenable to change.	27.3	48.1	24.6	51.3 (23.6)
**Items not included in any factor**				
2 The majority of depression seen in general practice originates from patients' recent misfortunes.	27.8	56.1	16.0	54.6 (20.1)
3 Most depressive disorders seen in general practice improve without medication.	6.4	39.6	54.0	35.1 (19.9)
4 An underlying biochemical abnormality is at the basis of severe cases of depression.	38.5	42.2	19.3	56.2 (24.1)
5 It is difficult to differentiate whether patients are presenting with unhappiness or a clinical depressive disorder that needs treatment.	43.9	40.1	16.0	59.0 (22.8)
9 I feel comfortable in dealing with depressed patients' needs.	0	16.0	84.0	19.4 (14.0)
11 Becoming depressed is a natural part of being old.	10.2	40.1	49.7	36.0 (23.3)
12 The practice nurse could be a useful person to support depressed patients.	67.4	28.3	4.3	72.7 (19.2)
18 Antidepressants usually produce a satisfactory result in the treatment of depressed patients in general practice.	41.7	45.5	12.8	59.1 (20.5)
20 If psychotherapy were freely available, this would be more beneficial than antidepressants for most depressed patients.	27.3	56.7	16.0	53.3 (21.6)

Each item score and factor score was divided for summary presentation: disagree (0-33.3), neutral (33.4-66.6), agree (66.7-100).

We calculated the mean score of items included in each factor derived from the factor analysis and defined this as the “factor score.” If item loadings were negative values, the subtracted values from 100 were used in the calculation.

None of the non-psychiatric doctors agreed with “*I feel comfortable in dealing with depressed patients' needs*” (Item 9). Similarly, items with “disagree” scores from more than 50 % of the non-psychiatric doctors were the following: “*Most depressive disorders seen in general practice improve without medication*” (Item 3), “*Becoming depressed is a way that people with poor stamina deal with life difficulties*” (Item 7), and “*There is little to be offered to those depressed patients who do not respond to what GPs do*” (Item 14).

### Exploratory analysis of factor structure

**Figure 1:**
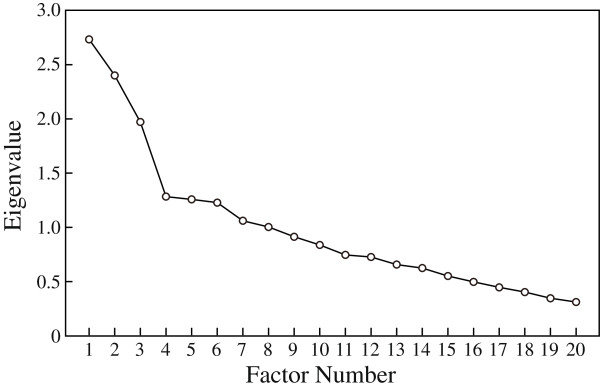
Scree Plot.

We supplementarily performed an exploratory factor analysis to extract major factors of the attitude. Eigenvalues (>1) from our exploratory factor analysis suggested an eight-factor solution. The scree plot from our exploratory factor analysis limited the number of major factors to three (Figure 1). Eight items (Items 2, 3, 4, 5, 11, 12, 18, and 20) that had a weak loading (<0.35) and one item (Item 9) with a low corrected item-total correlation value were excluded. The remaining 11 items had optimal loadings for one of three factors (see Table 4). The factor solution produced using an oblique rotation (promax) was similar to the three-factor structure (data not shown). Based on the content of included items, Factor I (Items 13, 17, and 19) was labeled “*Depression should be treated by psychiatrists,*” Factor II (Items 1, 14, 15, and 16) was labeled “*Pessimism regarding depression treatments,*” and Factor III (Items 6, 7, 8, and 10) was labeled “*Prejudice regarding depression etiology and pathology.*”

**Table 4:** Factor loadings by varimax rotation

**Item No.**	**Factor**		
	**I**	**II**	**III**
19	**0.882**	0.082	0.036
17	**0.601**	0.038	0.074
13	**0.394**	0.147	0.082
15	-0.153	**-0.611**	0.155
14	0.137	**0.538**	0.261
16	-0.024	**0.477**	0.117
1	-0.091	**-0.461**	0.019
7	-0.131	0.218	**0.653**
8	0.033	0.055	**0.484**
10	0.158	0.024	**0.432**
6	0.163	-0.211	**0.398**

Three factors accounted for 34.3 % of the total variance (Factor I: 12.9 %, Factor II: 11.2 %, and Factor III: 10.2 %). The Cronbach’s alpha value was 0.61 for the 11-item as a whole, and 0.65, 0.60, and 0.54 for Factors I, II, and III, respectively.

Mean scores of items included in each factor were also categorized into disagree, neutral, or agree. Fifty-four percent of non-psychiatric doctors agreed with items included in Factor I. More than half of non-psychiatric doctors were neutral on items in Factors II and III (54.5 % and 77.0 %, respectively; Table 3).

### Fitness of Japanese non-psychiatric doctors’ attitudes to the GPs’ factor models in the UK

As subanalysis, we determined the degree of fit between the present data for Japanese non-psychiatric doctors and two models derived from two independent surveys of GPs in the UK [7,9] using confirmatory factor analysis. Confirmatory factor analysis using a three-factor model of GPs in Glasgow which had been reported by Ross et al. [9], was unable to estimate the parameters, because the minimization was unsuccessful. Meanwhile, each fit index of a four-factor model reported by Botega et al. [7] was following: chi-square = 329.0, GFI = 0.824, AGFI = 0.761, CFI = 0.446, RMSEA = 0.101, and AIC = 409.0, which were worse than in the confirmatory factor analysis to examine the degree of fit to the Japanese three-factor model described above (chi-square = 110.3, GFI = 0.911, AGFI = 0.856, CFI = 0.754, RMSEA = 0.095, and AIC = 160.3).

## Discussion

This is the first report in Japan to present the attitudes of non-psychiatric doctors towards depression using the DAQ. Most non-psychiatric doctors agreed with Factor I, “*Depression should be treated by psychiatrists,*” which contained the items “*Working with depressed patients is heavy going*” (Item 13), “*If depressed patients need antidepressants, they are better off with a psychiatrist than with a general practitioner*” (Item 17), and “*Psychotherapy for depressed patients should be left to a specialist*” (Item 19). This suggests that most non-psychiatric doctors considered depression care to be beyond the scope of their duties.

In Japan, doctors have specialties and their role is to treat illnesses within their specialty. Given that depression is prevalent even in specialty clinics for physical illness [6], non-psychiatric doctors need to appropriately manage depressed patients. However, there is no model for depression care in Japan, such as the stepped-care model in the UK National Institute for Clinical Excellence (NICE) guidelines, which recommends that GPs and other members of the primary care team identify depressed patients and are actively involved in depression management [16]. Non-psychiatric doctors in Japan may think that depression should be treated by psychiatrists because there is no clear definition of their role in depression care in the Japanese medical system.

None of the non-psychiatric doctors in this study were comfortable dealing with the needs of depressed patients (Item 9). Furthermore, more than 50 % of the non-psychiatric doctors thought that the number of depressed patients has increased (Item 1). These results suggest that depression care is becoming an increasingly heavy burden for non-psychiatric doctors in Japan. It is of interest that most of the non-psychiatric doctors thought that nurse support was useful in depression care (Item 12): this suggests that cooperation between non-psychiatric doctors and other professionals, such as nurses and psychiatrists, can promote depression care. Collaborative and stepped-care models [16,17], in which various professionals cooperate with and provide support to non-psychiatric doctors, may be candidates for appropriate depression care models in Japan.

The non-psychiatric doctors in this study were likely to recognize the importance of depression care. However, they considered this to be beyond their role. This suggests that targeted educational interventions must address motivation for non-psychiatric doctors to play a role in depression care. Promoting their self-efficacy may help motivate them and facilitate their participation in caring for depressed patients. At the same time, a system that reduces the additional burden on non-psychiatric doctors may remove their implicit hesitation to perform screenings to identify depressed patients; this may be achieved by easier referral and improved collaboration with psychiatrists, and by developing the role of nurses in this area.

Use of the DAQ enables us to discuss differences between the attitudes of non-psychiatric doctors in Japan and those of GPs in the UK [7]. In Japan, many non-psychiatric doctors agreed with “*If depressed patients need antidepressants, they are better off with a psychiatrist than with a general practitioner*” (Item 17), whereas British GPs strongly disagreed with this item. This underscores the notion that Japanese non-psychiatric doctors do not recognize depression care as their role, whilst British GPs do. This difference may be due to differences in medical systems, such as the primary care system, between Japan and the UK. Japanese non-psychiatric doctors may also lack confidence in treating depressed patients by themselves, and prefer to refer patients to professionals, such as psychiatrists.

Many non-psychiatric doctors in Japan disagreed with “*Most depressive disorders seen in general practice improve without medication*” (Item 3), although British GPs generally agreed with this item [7]. Japanese non-psychiatric doctors may think medication is essential for treating depressed patients, whilst British GPs may be familiar with approaches other than antidepressants, such as cognitive behavioral therapy. Differences in knowledge about clinical outcomes of depression and effective treatment modalities may explain the difference in results for Item 3.

We compared the Japanese three-factor model derived in the present study with that of the three-factor model derived from factor loading using Glasgow GPs’ attitudes reported in a previous study [9]. The model derived from Glasgow study did not fit the Japanese non-psychiatric doctors’ attitude. It is suggested that Japanese non-psychiatric doctors’ attitude would be different from GPs in Glasgow. Similarly, we compared the Japanese three-factor model with that of the four-factor model derived from factor loading using other British GPs’ attitudes reported in a previous study [7]. Factor I derived from Japanese non-psychiatric doctors (Japanese Factor I), “*Depression should be treated by psychiatrists,*” shared two items (13 and 19) with Factor II derived from British GPs (British Factor II), “*Professional unease.*” Likewise, Japanese Factor III, “*Prejudice regarding depression etiology and pathology,*” shared two items (8 and 10) with British Factor III, “*Inevitable course of depression,*” which also included Item 17, *“If depressed patients need antidepressants, they are better off with a psychiatrist than with a general practitioner*”.

British Factor I, “*antidepressants/psychotherapy,*” included variables related to the role and relative effectiveness of antidepressants and psychotherapy in depression treatment, and included Items 3, 4, 7, 16, 18, and 20. There was no similar factor in the Japanese factor model, and items 3, 4, 18, and 20 were not included in any of the factors derived from Japanese non-psychiatric doctors. It appears that Japanese non-psychiatric doctors might not share a concept similar to that expressed by British Factor I, which may indicate that Japanese non-psychiatric doctors have limited knowledge of the roles and effectiveness of different depression treatments. Educational opportunities will be needed to promote the acquisition of knowledge on antidepressants and psychotherapy.

The present study has the following limitations. First, the sampling process, sample size, and response rate may have biased the results. Therefore, the generalizability is a major limitation. Second, we used the DAQ, which was developed in the UK where a medical system and culture are different from Japan. Third, there may be limitations with the validity of the DAQ as shown by various factor structures previously reported [15]. In addition, the three major factors obtained in this study accounted for only 34.3 % of the total variance. A questionnaire tailored to the Japanese medical system and culture may need to be developed in the future.

## Conclusion

The Japanese non-psychiatric doctors surveyed in this study believed that depression should be treated by psychiatrists. Underlying this attitude may be the lack of recognition of the necessity and motivation to participate in depression care. It is suggested that educational programs or guidelines for depression care developed in other countries such as the UK are not directly adaptable for Japanese non-psychiatric doctors. To improve depression care in Japan, it will be crucial to develop and implement focused interventions that motivate non-psychiatric doctors to play a role in depression care and to educate them about possible roles they can play.

## Competing interests

The authors declare that they have no competing interests.

## Authors’ contributions

All authors have read and approved the final version of the manuscript. MI was the principal investigator and developed the original idea for the study. MK, RS, FI, YW, MY, MI, MH and AM designed the study. TO, MK, MY, and MI analyzed data, and all authors discussed and prepared the manuscript. AM was a supervisor.

## Supplementary Material

Additional file 1 **Depression Attitude Questionnaire (DAQ)** 日本語版 Click here for file
