# Supplementary material for: Attitudes toward depression among Japanese non-psychiatric medical doctors: a cross-sectional study
Source: BMC Res Notes. 2012 Aug 16;5:441. doi: 10.1186/1756-0500-5-441 (PMC3434090; doi:10.1186/1756-0500-5-441)
Supplement: Additional file 1 — Depression Attitude Questionnaire (DAQ) 日本語版 [file 1756-0500-5-441-S1.pdf]

Depression Attitude Questionnaire (DAQ) 日本語版

この質問表には、うつ病に関する様々な意見を表した文章が含まれています。それぞれの文章の下に、「強く反対」を示す一端から「強く同意」を示す一端を結ぶ直線が描かれています。あなたの日々の臨床を最も反映する度合いは、下の直線でいえばどのあたりを示すかお答え下さい。例を参考に線上の一点を↓で表してください。

以下で使用する「一般診療科」という用語については、あなたの医療専門分野と置き換えてお答え下さい。

例： プライマリ・ケアの仕事にはうつ病患者への対応が含まれている

強く反対 ――――――――――――――↓――――― 強く同意

（上記は、反対というよりも、同意を示していますが、あいまいさを含む回答です。）

1. 過去5年間に、私は、うつ病症状のある患者数の増加を目の当たりにしてきた

強く反対 ―――――――――――――――――――― 強く同意

2. 一般診療科におけるうつ病の大多数は、患者の最近の不幸な出来事に起因する

強く反対 ―――――――――――――――――――― 強く同意

3. 一般診療科で遭遇するうつ病の大部分は、薬物療法なしに改善する

強く反対 ―――――――――――――――――――― 強く同意

4. 重症うつ病の基盤として、生化学的な異常が横たわる

強く反対 ―――――――――――――――――――― 強く同意

※　次のページへ続きます

※　「一般診療科」という用語については、あなたの医療専門分野と置き換えてお答え下さい。

5. 患者の症状が、単に不幸な状態か、それとも治療を必要とする臨床的なうつ病であるのかを区別するのは困難である

強く反対 ―――――――――――――――――――――――― 強く同意

6. うつ病は、心理的な原因によるうつ病と生化学的機序が原因のうつ病の二つの主な群に区別することができる

強く反対 ―――――――――――――――――――――――― 強く同意

7. うつ病になるということはスタミナに乏しい人々が人生の困難に対処する方法だ

強く反対 ―――――――――――――――――――――――― 強く同意

8. うつ病患者は、他の人々と比較して、幼少の時に剥奪（必要なものが欠如、喪失していること、または与えないこと）を経験していることが多い

強く反対 ―――――――――――――――――――――――― 強く同意

9. うつ病患者のニーズを扱うことは気楽なことだ

強く反対 ―――――――――――――――――――――――― 強く同意

10. うつ病は、変化に対応できないという患者の特徴的な反応を反映したものだ

強く反対 ―――――――――――――――――――――――― 強く同意

11. うつ病になるのは老いていく中で自然なことだ

強く反対 ―――――――――――――――――――――――― 強く同意

12. 看護師は、うつ病患者の支援に役立つ人となりうる

強く反対 ―――――――――――――――――――――――― 強く同意

※　次のページへ続きます

※　「一般診療科」という用語については、あなたの医療専門分野と置き換えてお答え下さい。

13. （あなたの日々の臨床において）うつ病患者と共に取り組むのは重いことだ

強く反対 ———————————————————— 強く同意

14. 一般診療科の治療に反応しないうつ病患者に提供されるべきことは少しもない

強く反対 ———————————————————— 強く同意

15. うつ病患者の面倒をみるために時間を費やすことは、報いのあることだ

強く反対 ———————————————————— 強く同意

16. うつ病患者に精神療法は失敗しがちだ

強く反対 ———————————————————— 強く同意

17. もし、うつ病患者が抗うつ薬を必要とするなら、一般診療科医よりも精神科医が診るほうが良い

強く反対 ———————————————————— 強く同意

18. 抗うつ薬は、うつ病患者の治療に、通常、満足いく結果を生じる

強く反対 ———————————————————— 強く同意

19. うつ病患者に対する精神療法は、専門家に任せるべきだ

強く反対 ———————————————————— 強く同意

20. もし、精神療法が制約なく利用可能なら、ほとんどのうつ病患者にとって、抗うつ薬より有益だろう

強く反対 ———————————————————— 強く同意

【出典】Botega N, Mann A, Blizard R, Wilkinson G: General practitioners and depression-first use of the depression attitude questionnaire. *Int J Methods Psychiatr Res* 1992, 2:169-180.

DAQ 日本語版は原著者より許諾を得て翻訳しています。

日本語版作成者：稲垣正俊（minagaki@ncnp.go.jp）
（独）国立精神・神経医療研究センター 精神保健研究所 自殺予防総合対策センター

調査研究で使用される場合には上記へご連絡ください。
